# Impact of School Closure Due to COVID-19 on the Social-Emotional Skills of Japanese Pre-school Children

**DOI:** 10.3389/fpsyt.2021.739985

**Published:** 2021-10-21

**Authors:** Satomi Doi, Keitaro Miyamura, Aya Isumi, Takeo Fujiwara

**Affiliations:** ^1^Department of Global Health Promotion, Tokyo Medical and Dental University (TMDU), Tokyo, Japan; ^2^Japan Society for the Promotion of Science, Tokyo, Japan

**Keywords:** COVID-19, pre-school, social-emotional skills, school activity, school closure

## Abstract

**Objective:** This study examines the impact on the social-emotional skills of Japanese pre-school children from downsizing of school activities in conjunction with voluntary school closures due to the first wave of COVID-19, in 2020.

**Methods:** Participants included 32 children aged 4–5 years old from three pre-schools in Tokyo, Japan, where strict lockdown was not implemented and voluntary school closure was recommended. Child social-emotional skills was assessed by classroom teachers using Devereux Student Strengths Assessment mini (DESSA-mini) three times: November 2019, January 2020 (before the COVID-19), and March 2020 (during the first COVID-19 wave). All pre-schools implemented voluntary school closures from March 2nd, and two schools (school A and B) canceled school recitals, while one school (school C) allowed for it to be held on March 4th, with precautions in place to prevent the spread of infection. Repeated measures ANOVA were performed to examine the difference between the T scores of the DESSA-mini three pre-schools before and during the COVID-19 pandemic.

**Results:** In school C, children showed stable T scores of the DESSA-mini, whereas children in school A and B showed lower T scores of the DESSA-mini during COVID-19 than before it started. The interaction effects between time and pre-schools were found (*F* = 7.05, *p* < 0.001).

**Conclusion:** Our findings suggest that school recitals in pre-schools were important to maintaining children's social-emotional skills during the COVID-19 pandemic.

## Introduction

The first outbreak of the coronavirus disease in 2019 (COVID-19) impacted pre-school children in many countries in various ways, including school closures and the downsizing of activities such as school recitals to prevent the spread of infection (1). A growing number of studies have examined the short and long-term impacts of school closure on mental health of pre-school children and their caregivers. Increased behavioral problems (2), decreased physical activities (2, 3), weight gain (4), impaired quality of sleep (3, 5), and increased screen time (3) have been found among pre-school children after the closure of schools.

Even though there are studies that summarize possible adverse impacts of pre-school closure on children (6–8), to the best of our knowledge, few studies investigate the impact of school closures by comparing schools which canceled school activities, to schools which implemented school activities despite COVID-19. Japan is a unique country, in that a severe lockdown was not implemented, and pre-school closure was only recommended by the government, not mandatory. In addition, implementation of school activities relied on the discretion of individual pre-school principals. In the Japanese system of pre-school, child aged from 0 to 5 years can go to pre-school with no charge (not always). In 2020, approximately 50% of children attended pre-school. Almost all pre-schools in Tokyo, Japan conduct a school recital (e.g., dancing, singing, and drama activities) as school activities, which is held once per year (9). The aims of the school recital are not only to develop child's creativeness but also to show child's growth for caregivers (9). Thus, the school recital in Japanese pre-school has an important role for children and caregivers, which is also a major event for teachers.

Fortunately, we were able to make contact with three schools that agreed to take part in our research; two of which canceled scheduled school recitals, and one that allowed for the recital as scheduled, on March 4th. In all three schools we were able to assess the children's mental health before and during the first wave of COVID-19. Thus, by using this precious data, we could evaluate the impact of school closures by considering the mental health status of the children before COVID-19, and comparing the schools which canceled school activities with the school which allowed for regular school activities to continue.

Furthermore, to date, previous studies have focused on behavioral changes related to life style such as physical activity and screen time (2–5) and negative aspects of mental health such as behavioral problems (2). Little is known about positive aspects of mental health such as the development of social-emotional skills, which is defined as “skills to understand and manage emotions, set and achieve positive goals, feel and show empathy for others, establish and maintain positive relationships, and make responsible decisions” (10). The OECD (11) suggests that the development of children's social-emotional competencies is required because it has a significant role within well-being, life satisfaction, healthy life style, and academic success. In Japan, the government guideline for pre-school education also address developing social-emotional skills. It may become more important for children to have the ability of adaptation for unexpected situations, due to the pandemic of COVID-19, than ever before.

This study examines the impact of school closure due to the first wave of COVID-19, in early 2020, on social-emotional skills among Japanese pre-school children.

## Methods

### Participants

Participants included 32 children aged 4–5 years old from three pre-schools in Tokyo, Japan, where strict lockdown was not implemented and voluntary school closure was recommended. All pre-schools implemented voluntary school closures from March 2nd to May 30th, 2020, and two schools (school A and B) canceled school recitals during voluntary school closure periods, but one school (school C) had a recital on March 4th, with infection prevention measures in place.

### Measures

Child's social-emotional skills was assessed by class teachers using Devereux Student Strengths Assessment-Mini (DESSA-mini) (12–14), which is one of the major assessment tools of child's social-emotional skills and is applicable to children aged 5–14 years old. The DESSA-mini consists of 8 items assessed on a five-point Likert scale (from 0 = ”never” to 4 = ”very frequently”) and has a high internal consistency (Cronbach α = 0.919). Educators can rate children's social emotional competence during a 4 week period, within 1 min. Based on raw score sum which ranges from 0 to 32, a T score can be calculated. Furthermore, we can obtain three categories using T score: “strength” (T score > 60), “typical” (41 < T score < 59), and “need for instruction” (T score <40). In this study, the Japanese version of DESSA-mini was developed with permission of the developer of the original English version of DESSA-mini. According to the translation policy provided by the developer of the original English version of DESSA-mini, two authors who are familiar with child development and psychology independently translated the items from the original English to the Japanese. After completing the initial translations, translators discussed any inconsistencies. Two other translators independently back-translated a final translation and compared and resolved inconsistencies. All drafts of the translation and back-translation were sent to the original developer. The Cronbach α of this study was 0.919.

### Procedure

In the three pre-schools that participated in this study, there were two class teachers per/class. The main teachers were in-charge of assessing each child's social-emotional skills using the DESSA-mini at three times: November 2019 (Time 1), January 2020 (Time 2) (before the COVID-19), and March 2020 (Time 3, during the first wave of COVID-19). Prior to the first assessment, we held orientation meetings for teachers in each pre-school in order to explain the aim of this study and how to assess child's social-emotional skills using the DESSA-mini questionnaire. After the orientation meetings, the DESSA-mini questionnaires were distributed to pre-schools. The main teachers per class completed the DESSA-mini questionnaires for all children in their own class without discussing with other teachers. The main teacher completed child's name, sex, date of birth, age, teacher's name, relationship with a child, the date of response to the questionnaire, pre-school's name, class's name, and 8 items related to child's social-emotional skills. Additionally, they calculated raw score sums and T scores for each child and filled the percentile and category using T score (i.e., strength, typical, or need). In Schools A and B conducted the assessment on November 28th, while school C's assessment was on November 29th, 2019. Schools A and B conducted the 2nd assessments on January 24th, 2020, and school C's assessment was on the 31st. The third assessment was held by schools A, B, and C on March 4th, 27th, and 30th, 2020, respectively. After completing each assessment, pre-schools sent the questionnaires to our office. In Japan, the number of infections increased from March 2020. The Japanese government announced a state of emergency for all prefectures from April 16th to May 25th 2020, in which people were asked to exercise self-restraint. In Tokyo, where infection was widely spread, the state of emergency was declared from April 4th to May 25th (15, 16). During the first wave of the COVID-19 pandemic, the number of infections were fewer than 30 per day in Japan (16).

### Statistical Analysis

First, we performed a repeated measures mixed model to examine the association of time (i.e., Time 1: November 2019, Time 2: January 2020, and Time 3: March 2020), schools (i.e., School A, School B, and School C), and the interaction between time and school with T score of the DESSA-mini. In this analysis, the reference time was Time 2 (January 2020 which was before the first wave of COVID-19) in order to compare the differences between before and during the COVID-19, mainly. Second, repeated measures ANOVA was also performed to examine the difference of T scores of the DESSA-mini three schools, at three times. Child's sex was adjusted in the first and second analyses. Third, a Kruskal-Wallis test, which is one-way ANOVA on ranks and a non-parametric method, was conducted due to small sample size as the sensitive analysis. We performed the Kruskal-Wallis tests to compare the mean of the DESSA-mini score by time and school, respectively. According to the results of the Kruskal-Wallis test, a Dunn's test which is pairwise Mann-Whitney tests with Bonferroni correction was also performed. All analyses were conducted using STATA 15.0.

## Results

Table 1 shows the characteristics of the participants in this study. Participants included 22 males (68.7%) and 10 females (31.3%). Their mean age was 60.59 (±3.58) months. Among school A, B, and C, the percentages of females were 33.3, 27.3, and 33.3% respectively (no statistical differences between percentages of each sex, *p* = 0.831). The mean ages were 61.89 (±2.74), 60.36 (±3.55), and 59.83 (±3.97), respectively (no statistical differences in the mean ages, *p* = 0.069).

**Table 1:** Characteristics of sample in this study (*n* = 32).

	**Total (*****n*** **=** **32)**		**School A (*****n*** **=** **9)**		**School B (*****n*** **=** **11)**		**School C (*****n*** **=** **12)**		
	***n*** **or mean**	**% or SD**	***n*** **or mean**	**% or SD**	***n*** **or mean**	**% or SD**	***n*** **or mean**	**% or SD**	***p***
**Child's sex**									
Male	22	68.7	6	66.7	8	72.7	8	66.7	0.831
Female	10	31.3	3	33.3	3	27.3	4	33.3	
Mean age (month)	60.59	3.58	61.89	2.74	60.36	3.55	59.83	3.97	0.069

*SD, standard deviation*.

In Table 2, the mean T scores for time and school and the results of repeated measures mixed model were shown. Among all participants, the mean T score of the DESSA-mini by time were 45.16 (SD = 8.78) at November 2019 (Time 1), 51.52 (SD = 8.27) at January 2020 (Time 2), and 49.65 (SD = 6.99) at March 2020 (Time 3). According to the main effect of time (Model 1), the mean T score of the DESSA-mini at November 2019 (Time 1) was lower than in January 2020 (Time 2) (coefficient = −5.93; 95% CI = −7.89 to −3.96). The mean T score of the DESSA-mini by schools were 52.89 (SD = 6.22) in school A, 43.13 (SD = 8.23) in school B, and 50.44 (SD=7.55) in school C. Compared to school A, the mean T score of the DESSA-mini in school B was significantly lower (coefficient = −9.70; 95% CI = −14.51 to −4.89).

**Table 2:** Results of repeated measures mixed model (*n* = 94).

		**T score**	**Model 1**	**Model 2**
		**Mean (SD)**	**Coefficient (95% CI)**	**Coefficient (95% CI)**
Time	Time 1 (November 2019)	45.16 (8.78)	−5.93 (−7.89 to −3.96)	−3.44 (−6.52 to −0.37)
	Time 2 (January 2020)	51.52 (8.27)	Ref	Ref
	Time 3 (March 2020)	49.65 (6.99)	−1.87 (−3.84 to 0.10)	−4.89 (−7.96 to −1.81)
School	A	52.89 (6.22)	Ref	Ref
	B	43.13 (8.23)	−9.70 (−14.51 to −4.89)	−7.97 (−13.32 to −2.63)
	C	50.44 (7.55)	−2.44 (−7.13 to 2.24)	−4.25 (−9.43 to 0.93)
Time × School	Time 1 (November 2019) × School B			−7.26 (−11.48 to −3.03)
	Time 3 (March 2020) × School B			2.59 (−1.65 to 6.83)
	Time 1 (November 2019) × School C			−0.22 (−4.29 to 3.85)
	Time 3 (March 2020) × School C			5.64 (1.57 to 9.71)

*Child's sex was adjusted*.

**Figure 1:**
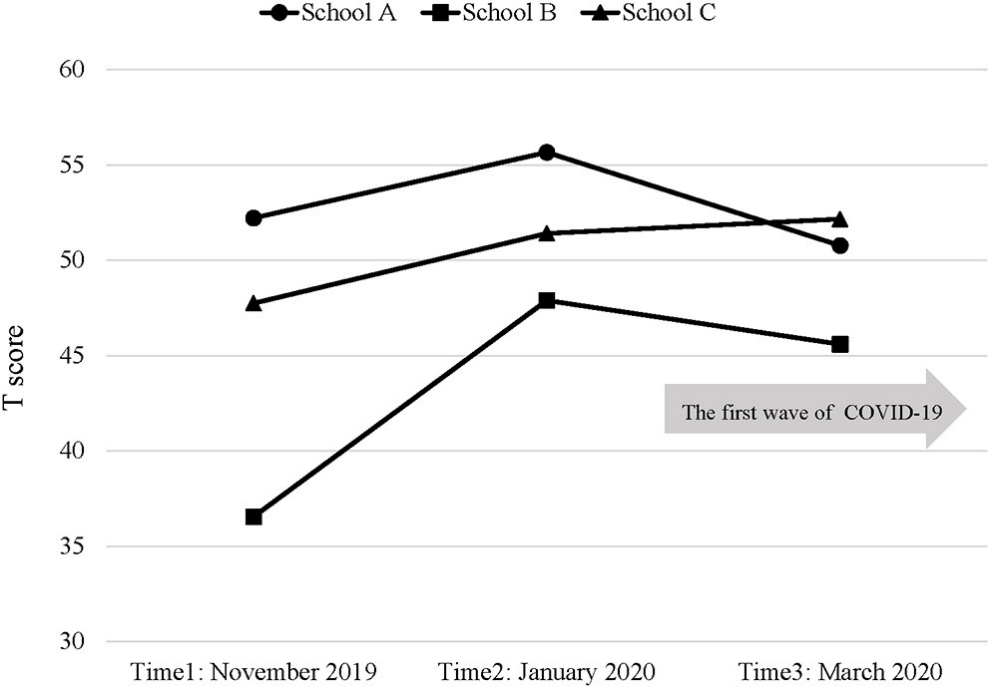
T scores of the DESSA-mini.

Children in school A and B showed lower T scores of the DESSA-mini during the COVID-19 pandemic than before it: 52.22 (SD = 6.12) at November 2019 (Time 1), 55.67 (SD = 6.24) at January 2020 (Time 2), and 50.78 (SD = 5.93) at March 2020 (Time 3) in school A; 36.55 (SD = 5.43) at November 2019 (Time 1), 47.90 (SD = 8.45) at January 2020 (Time 2), and 45.60 (SD = 6.06) at March 2020 (Time 3) in school B. In contrast, in school C, children showed stable T scores of the DESSA-mini: 47.75 (SD = 6.40) at November 2019 (Time 1), 51.42 (SD = 8.58) at January 2020 (Time 2), and 52.17 (SD = 7.40) at March 2020 (Time 3) (Figure 1). Supplementary Table 1 shows T score, raw score sum, each item score of the DESSA-mini, and the numbers of categories. In Model 2, the interaction term between time and school was added. Compared to T score at January 2020 (Time 2, which was before the COVID-19), the T score of the DESSA-mini at March 2020 (Time 3, which was during the COVID-19) was significantly lower (coefficient = −4.89; 95% CI = −7.96 to −1.81). Moreover, the T score of the DESSA-mini at November 2019 (Time 1) was significantly lower compared to that at January 2020 (Time 2) (coefficient = −3.44; 95% CI = −6.52 to −0.37). In terms of the effect of school, children in school B showed a lower T score than those in school A (coefficient = −7.97; 95% CI = −13.32 to −2.63). Similarly, repeated measures ANOVA also showed that the effects of time (*F* = 24.37, *p* < 0.001) and school (*F* = 8.41, *p* < 0.001) were significant in Table 3. Furthermore, we found the interaction effects between time and pre-schools (*F* = 7.05, *p* < 0.001).Children in school A and B showed lower T scores of the DESSA-mini during the COVID-19 pandemic than before it: 52.22 (SD = 6.12) at November 2019 (Time 1), 55.67 (SD = 6.24) at January 2020 (Time 2), and 50.78 (SD = 5.93) at March 2020 (Time 3) in school A; 36.55 (SD = 5.43) at November 2019 (Time 1), 47.90 (SD = 8.45) at January 2020 (Time 2), and 45.60 (SD = 6.06) at March 2020 (Time 3) in school B. In contrast, in school C, children showed stable T scores of the DESSA-mini: 47.75 (SD = 6.40) at November 2019 (Time 1), 51.42 (SD = 8.58) at January 2020 (Time 2), and 52.17 (SD = 7.40) at March 2020 (Time 3) (Figure 1). Supplementary Table 1 shows T score, raw score sum, each item score of the DESSA-mini, and the numbers of categories. In Model 2, the interaction term between time and school was added. Compared to T score at January 2020 (Time 2, which was before the COVID-19), the T score of the DESSA-mini at March 2020 (Time 3, which was during the COVID-19) was significantly lower (coefficient = −4.89; 95%CI = −7.96 to −1.81). Moreover, the T score of the DESSA-mini at November 2019 (Time 1) was significantly lower compared to that at January 2020 (Time 2) (coefficient = −3.44; 95% CI = −6.52 to −0.37). In terms of the effect of school, children in school B showed a lower T score than those in school A (coefficient = −7.97; 95% CI = −13.32 to −2.63). Similarly, repeated measures ANOVA also showed that the effects of time (*F* = 24.37, *p* < 0.001) and school (*F* = 8.41, *p* < 0.001) were significant in Table 3. Furthermore, we found the interaction effects between time and pre-schools (*F* = 7.05, *p* < 0.001).

**Table 3:** Results of repeated measures ANOVA (*n* = 94).

	***F***	***p*** **-value**
Time	24.37	<0.001
School	8.41	0.001
Time × School	7.05	<0.001

*ANOVA, analysis of variance and covariance*.

*Child's sex was adjusted*.

As a sensitive analysis, the results of a Kruskal-Wallis test showed a significant difference in the mean T score of the DESSA-mini by time (χ^2^ = 9.29, *p* = 0.009, df = 2). Pairwise comparison showed that the mean T score of the DESSA-mini at November 2019 (Time 1) was lower than in January 2020 (Time 2) (*p* < 0.005). The results also showed a significant difference in the mean T score of the DESSA-mini by schools (χ^2^ = 20.67, *p* < 0.001, df = 2). Pairwise comparison showed that that the mean T score of the DESSA-mini in school B was significantly lower than school A (*p* < 0.001) and school B (*p* < 0.005). We found similar results with those of the ANOVA.

## Discussion

This study found that social-emotional skills in pre-school children increased from November 2019 to January 2020, which was before the first wave of COVID-19 in Japan, and in March 2020, during COVID-19. Furthermore, we found the interaction effects between time and schools on social-emotional skills. That is, children in school C, which allowed for the school recital as planned on March 4th, during the first wave of COVID-19, showed a stable T score of the DESSA-mini, whereas children in schools A and B, which canceled the school recital due to the COVID-19 pandemic, showed a lower T score of the DESSA-mini during the COVID-19 than before COVID-19. Therefore, our findings indicate that the school recital, with infection prevention measures, was important to keep children's social-emotional skills, under the circumstances of the COVID-19 pandemic.

The changes in social-emotional skills among children can be attributed to the activities of the pre-schools (i.e., the school recital) rather than school closures because schools that participated in our survey did not implement school closures due to COVID-19. The novelty of this study is that this survey could evaluate the impact of school activities during the first COVID-19 wave, using date before the COVID-19 pandemic, and comparing both schools that allowed for a school recital and those that did not. The conducting of a school recital might not cause lifestyle changes such as decreased physical activity and increased screen time which was found in the previous studies that examined the impacts of school closure under the pandemic of COVID-19 (8, 17, 18). Rather than child's lifestyle changes, the implementation of a school recital might lead to increased opportunities to develop a child's skills related to social-emotional skills. Brooks (19) emphasizes the importance of building a child's social-emotional skills by using the opportunities at school, which indicates that maximizing activity opportunities that children can participate in is meaningful in promoting social-emotional skills. The implementation of a school recital, which includes preparation time, could create the opportunities to develop a child's skills which are critical factors to build social-emotional skills, such as skills in communication, control of their emotions, and problem solving (20–22).

Another possible factor for social-emotional skills is the change of parenting behaviors, because, although these pre-schools were not closed, it was recommended that children not attend school, which means the time spent with a parent or other caregiver at home would increase. A previous study in Singapore showed that parental perceived impact of COVID-19 was associated with increased harsh parenting and poor parent-child relationships via parenting stress (23). Thus, increased time spent at home due to COVID-19 might lead to a change in the parent-child relationship. Additionally, the relationship between school and caregivers, which is an important factor for developing a child's social-emotional skills (19), might be promoted via the implementation of school activities. Further study to examine the impacts of the implementation of school activities on not only children, but also caregivers is warranted.

The current study has the following limitations. First, the sample size was small even though this is precious data which allowed us to evaluate the impact of school closure by comparing schools that canceled school activities and a school which continued with school activities. Further study to examine the long-term impacts of COVID-19 and related school factors on child social-emotional skills is needed. Second, we only adjusted for child's sex in the analyses. Unfortunately, we could not assess family factors such as paternal mental health, parenting behaviors, and household income. Our findings need to be re-verified, including potential confounders using available data. Third, generalizability of the current study is limited because the number of infections and school impacts of COVID-19 varied between countries. However, there is a need to further explore the impacts of school closure on child social-emotional skills under different various situations. Fourth, we did not assess other positive mental health aspects such as prosocial behaviors assessed using the Strengths and Difficulties Questionnaire (SDQ) (24) even though this study focused on the changes of children's social-emotional skills. For example, combined assessment, in which caregiver assesses child's prosocial behaviors using the SDQ, may be helpful to figure out the changes in children's positive mental health broadly under the circumstances of the COVID-19 pandemic.

## Conclusions

In conclusion, our findings suggest that school closure might be associated with lower levels of social-emotional skills among pre-school children. However, we also indicate that school activities, with infection prevention measures in place, may be important in maintaining children's social-emotional skills, during the COVID-19 pandemic. Understandably, pre-schools and caregivers want to protect their children against infection. Nonetheless, we need to focus on and care about child's social-emotional skills during the long-term pandemic at the same time. These results may be helpful when deciding during times of emergency, like those of COVID-19, whether a school should close or continue regularly scheduled activities.

## Data Availability Statement

The data analyzed in this study is subject to the following licenses/restrictions: due to the nature of this research, participants of this study did not agree for their data to be shared publicly, so supporting data is not available. Requests to access these datasets should be directed to fujiwara.hlth@tmd.ac.jp.

## Ethics Statement

The studies involving human participants were reviewed and approved by the Institutional Review Board of the Tokyo Medical and Dental University (M2020-380). Written informed consent from the participants' legal guardian/next of kin was not required to participate in this study in accordance with the national legislation and the institutional requirements.

## Author Contributions

TF, AI, and SD designed the study and managed administration of the study, including the ethical review process. KM and SD analyzed data. SD drafted the manuscript. AI and TF provided critical comments on the manuscript related to intellectual content. All authors have read and approved the final manuscript.

## Funding

This study was supported by Grants-in-Aid for Scientific Research from the Japan Society for the Promotion of Science (JSPS KAKENHI Grant No. 21H04848) and 21K18294.

## Conflict of Interest

The authors declare that the research was conducted in the absence of any commercial or financial relationships that could be construed as a potential conflict of interest.

## Publisher's Note

All claims expressed in this article are solely those of the authors and do not necessarily represent those of their affiliated organizations, or those of the publisher, the editors and the reviewers. Any product that may be evaluated in this article, or claim that may be made by its manufacturer, is not guaranteed or endorsed by the publisher.

## References

[1] UNESCO (2020). Covid-19 Educational Disruption and Response. UNESCO. Available online at: https://en.unesco.org/covid19/educationresponse

[2] Tso WWY Wong RS Tung KTS Rao N Fu KW Yam JCS. Vulnerability and resilience in children during the COVID-19 pandemic. Euro Child Adolesc Psychiatry. (2020) 1–16. 10.1007/s00787-020-01680-8 33205284 PMC7671186

[3] Aguilar-Farias N Toledo-Vargas M Miranda-Marquez S Cortinez-O'Ryan A Cristi-Montero C Rodriguez-Rodriguez F. Sociodemographic predictors of changes in physical activity, screen time, and sleep among toddlers and preschoolers in chile during the COVID-19 pandemic. Int J Environ Res Public Health. (2021) 18:176. 10.3390/ijerph18010176 33383721 PMC7796176

[4] Takaku R Yokoyama I. What the COVID-19 school closure left in its wake: evidence from a regression discontinuity analysis in Japan. J Public Econ. (2020) 195:104364. 10.1016/j.jpubeco.2020.104364 33437102 PMC7791313

[5] Dellagiulia A Lionetti F Fasolo M Verderame C Sperati A Alessandri G. Early impact of COVID-19 lockdown on children's sleep: a 4-week longitudinal study. J Clin Sleep Med. (2020) 16:1639–40. 10.5664/jcsm.8648 32620188 PMC7970607

[6] Chat A Roy S. COVID-19 and its impact on education of preschool students in a developing nation: a viewpoint. SSRN. (2020) 3722505.

[7] Silverman M Sibbald R Stranges S. Ethics of COVID-19-related school closures. Can J Public Health. (2020) 111:462–5. 10.17269/s41997-020-00396-1 32767271 PMC7412780

[8] Rundle G Park Y Herbstman JB Kinsey EW Wang YC. COVID-19–related school closings and risk of weight gain among children. Obesity. (2020) 28:1008–9. 10.1002/oby.22813 32227671 PMC7440663

[9] Nakayama K. The concept of nursery teachers and kindergarten teachers for drama activities and the present situation of drama activities at daycare centers and kindergartens: via a questionnaire survey into public kindergartens and nurseries in the 23 Wards of Tokyo. J Grad Sch Home Econ Hum Life Sci. (2018) 24:161–71.

[10] Collaborative for Social and Emotional Learning (2015). CASEL Guide: Effective Social and Emotional Learning Programs. Available online at: http://secondaryguide.casel.org/casel-secondary-guide.pdf

[11] OECD (2015). Skills for Social Progress: The Power of Social and Emotional Skills. Paris: OECD Publishing. 10.1787/9789264226159-en

[12] Naglieri JA LeBuffe P Shapiro VB. Universal screening for social–emotional competencies: a study of the reliability and validity of the DESSA-mini. Psychol Sch. (2011) 48:660–71. 10.1002/pits.20586

[13] Naglieri JA LeBuffe PA Shapiro VB. Universal screening for socialemotional competencies: A study of the reliability and validity of the DESSA-mini.*Psychol Schools*. (2011) 48:660–71.

[14] Shapiro VB Kim BKE Robitaille JL LeBuffe PA. Protective factor screening for prevention practice: sensitivity and specificity of the DESSA-Mini. Sch Psychol Q. (2017) 32:449–64. 10.1037/spq0000181 27736121

[15] Fukase Y Ichikura K Murase H Tagaya H. Depression, risk factors, and coping strategies in the context of social dislocations resulting from the second wave of COVID-19 in Japan. BMC Psychiatry. (2021) 21:33. 10.1186/s12888-021-03047-y 33435930 PMC7802816

[16] Karako K Song P Chen Y Tang W Kokudo N. Overview of the characteristics of and responses to the three waves of COVID-19 in Japan during 2020-2021. BioScience Trends. (2021) 15:1–8. 10.5582/bst.2021.01019 33518668

[17] López-Bueno R López-Sánchez GF Casajús JA Calatayud J Tully MA Smith L. Potential health-related behaviors for pre-school and school-aged children during COVID-19 lockdown: a narrative review. Prev Med. (2020) 143:106349. 10.1016/j.ypmed.2020.106349 33271236 PMC7701882

[18] Guan H Okely AD Aguilar-Farias N Del Pozo Cruz B Draper CE. Promoting healthy movement behaviours among children during the COVID-19 pandemic. Lancet Child Adolesc Health. (2020) 4:416–8. 10.1016/S2352-4642(20)30131-0 32458805 PMC7190292

[19] Brooks JE. Strengthening resilience in children and youths: maximizing opportunities through the schools. Child Sch. (2006) 28:69–76. 10.1093/cs/28.2.69

[20] Farrell D Meyer AL White KS. Evaluation of responding in peaceful and positive ways (RIPP): a school-based prevention program for reducing violence among urban adolescents. J Clin Child Psychol. (2001) 30:451–63. 10.1207/S15374424JCCP3004_02 11708233

[21] Griffin KW Botvin GJ Nichols TR Doyle MM. Effectiveness of a universal drug abuse prevention approach for youth at high risk for substance use initiation. Prev Med. (2003) 36:1–7. 10.1006/pmed.2002.1133 12473419

[22] Pahl KM Barrett PM. The development of social-emotional competence in preschool-aged children: an introduction to the fun FRIENDS program. Austra J Guid Counsel. (2007) 17:81. 10.1375/ajgc.17.1.81

[23] Chung G Lanier P Wong PYJ. Mediating effects of parental stress on harsh parenting and parent-child relationship during coronavirus (COVID-19) pandemic in Singapore. J Fam Viol. (2020) 1–12. 10.1007/s10896-020-00200-1 32895601 PMC7467635

[24] Muris P Meesters C van den Berg F. The strengths and difficulties questionnaire (SDQ). Euro Child Adolesc Psychiatry. (2003) 12:1–8. 10.1007/s00787-003-0298-2 12601558

