# Supplementary material for: Impact of School Closure Due to COVID-19 on the Social-Emotional Skills of Japanese Pre-school Children
Source: Front Psychiatry. 2021 Oct 21;12:739985. doi: 10.3389/fpsyt.2021.739985 (PMC8566816; doi:10.3389/fpsyt.2021.739985)
Supplement: Supplementary file 1 [file Table_1.docx]

| Supplemental Table 1 Distribution of DESSA (n=32) | | | | |  |  |  |  |  |  |  |  |  |  |  |  |  |  |  |  |  |  |  |  |  |  |  | |  |  |  |  |  |  |  |  |
| --- | --- | --- | --- | --- | --- | --- | --- | --- | --- | --- | --- | --- | --- | --- | --- | --- | --- | --- | --- | --- | --- | --- | --- | --- | --- | --- | --- | --- | --- | --- | --- | --- | --- | --- | --- | --- |
|  | Total | | | | | | | |  | School A | | | | | | | |  | School B | | | | | | | | |  | School C | | | | | | | |
|  | Time 1 November 2019 (n=32) | |  | Time 2  January 2020 (n=31) | |  | Time 3 March 2020  (n=31) | |  | Time 1 November 2019 (n=9) | |  | Time 2  January 2020 (n=9) | |  | Time 3 March 2020  (n=9) | |  | Time 1 November 2019 (n=11) | |  | Time 2  January 2020 (n=10) | |  | Time 3 March 2020  (n=10) | |  | | Time 1 November 2019 (n=12) | |  | Time 2  January 2020 (n=12) | |  | Time 3 March 2020  (n=12) | |
|  | mean or n | SD or % |  | mean or n | SD or % |  | mean or n | SD or % |  | mean or n | SD or % |  | mean or n | SD or % |  | mean or n | SD or % |  | mean or n | SD or % |  | mean or n | SD or % |  | mean or n | SD or % |  | | mean or n | SD or % |  | mean or n | SD or % |  | mean or n | SD or % |
| T score | 45.16 | 8.78 |  | 51.52 | 8.27 |  | 49.65 | 6.99 |  | 52.22 | 6.12 |  | 55.67 | 6.24 |  | 50.78 | 5.93 |  | 36.55 | 5.43 |  | 47.90 | 8.45 |  | 45.60 | 6.06 |  | | 47.75 | 6.40 |  | 51.42 | 8.58 |  | 52.17 | 7.40 |
| Total score | 18.63 | 6.19 |  | 22.65 | 5.47 |  | 21.74 | 4.27 |  | 23.44 | 3.32 |  | 25.33 | 3.12 |  | 22.67 | 3.24 |  | 12.36 | 5.01 |  | 20.20 | 6.94 |  | 19.20 | 4.08 |  | | 20.75 | 3.74 |  | 22.67 | 4.89 |  | 23.17 | 4.43 |
| Item 1 | 2.09 | 0.89 |  | 2.61 | 0.92 |  | 2.48 | 0.77 |  | 2.67 | 0.71 |  | 3.33 | 0.71 |  | 2.56 | 0.73 |  | 1.36 | 0.67 |  | 1.90 | 0.74 |  | 2.20 | 0.63 |  | | 2.33 | 0.78 |  | 2.67 | 0.78 |  | 2.67 | 0.89 |
| Item 2 | 2.31 | 0.90 |  | 2.55 | 0.81 |  | 2.74 | 0.63 |  | 3.00 | 0.50 |  | 3.22 | 0.44 |  | 3.00 | 0.50 |  | 1.45 | 0.69 |  | 1.90 | 0.74 |  | 2.60 | 0.52 |  | | 2.58 | 0.67 |  | 2.58 | 0.67 |  | 2.67 | 0.78 |
| Item 3 | 2.19 | 1.00 |  | 2.52 | 0.96 |  | 2.61 | 0.62 |  | 3.11 | 0.33 |  | 3.56 | 0.53 |  | 2.89 | 0.33 |  | 1.36 | 0.67 |  | 1.90 | 0.74 |  | 2.40 | 0.52 |  | | 2.25 | 0.97 |  | 2.25 | 0.75 |  | 2.58 | 0.79 |
| Item 4 | 2.09 | 1.00 |  | 2.35 | 0.84 |  | 2.58 | 0.92 |  | 2.56 | 1.01 |  | 2.56 | 0.53 |  | 2.44 | 1.01 |  | 1.36 | 0.67 |  | 1.90 | 0.74 |  | 2.30 | 0.67 |  | | 2.42 | 0.90 |  | 2.58 | 1.00 |  | 2.92 | 1.00 |
| Item 5 | 2.34 | 0.90 |  | 3.06 | 0.85 |  | 2.81 | 0.75 |  | 2.89 | 0.60 |  | 3.00 | 0.71 |  | 2.89 | 0.60 |  | 2.00 | 1.15 |  | 2.30 | 0.82 |  | 1.36 | 0.67 |  | | 2.75 | 0.62 |  | 3.17 | 0.72 |  | 3.17 | 0.58 |
| Item 6 | 2.28 | 0.96 |  | 2.97 | 0.95 |  | 2.74 | 0.77 |  | 2.89 | 0.78 |  | 3.11 | 0.78 |  | 2.78 | 0.83 |  | 1.36 | 0.67 |  | 2.80 | 1.31 |  | 2.20 | 0.63 |  | | 2.67 | 0.65 |  | 3.00 | 0.74 |  | 3.17 | 0.58 |
| Item 7 | 2.40 | 1.07 |  | 3.23 | 0.88 |  | 2.87 | 0.72 |  | 3.11 | 1.05 |  | 3.22 | 0.83 |  | 3.00 | 0.87 |  | 1.36 | 0.67 |  | 3.20 | 1.23 |  | 2.50 | 0.53 |  | | 2.83 | 0.58 |  | 3.25 | 0.62 |  | 3.08 | 0.67 |
| Item 8 | 2.91 | 0.69 |  | 3.35 | 0.75 |  | 2.90 | 0.60 |  | 3.22 | 0.83 |  | 3.33 | 0.50 |  | 3.11 | 0.33 |  | 2.64 | 0.5 |  | 3.60 | 0.70 |  | 2.70 | 0.48 |  | | 2.92 | 0.67 |  | 3.17 | 0.94 |  | 2.92 | 0.79 |
| Category |  |  |  |  |  |  |  |  |  |  |  |  |  |  |  |  |  |  |  |  |  |  |  |  |  |  |  | |  |  |  |  |  |  |  |  |
| Strength | 3 | 9.4 |  | 5 | 16.1 |  | 2 | 6.4 |  | 2 | 22.2 |  | 2 | 22.2 |  | 1 | 11.1 |  | 0 | 0 |  | 1 | 10.0 |  | 0 | 0 |  | | 1 | 8.3 |  | 2 | 16.7 |  | 1 | 8.3 |
| Typical | 22 | 68.7 |  | 24 | 77.4 |  | 26 | 83.9 |  | 7 | 77.8 |  | 7 | 77.8 |  | 8 | 88.9 |  | 5 | 45.5 |  | 8 | 80.0 |  | 8 | 80.0 |  | | 10 | 83.3 |  | 9 | 75.0 |  | 10 | 83.3 |
| Need | 7 | 21.9 |  | 2 | 6.5 |  | 3 | 9.7 |  | 0 | 0 |  | 0 | 0 |  | 0 | 0 |  | 6 | 54.5 |  | 1 | 10.0 |  | 2 | 20.0 |  | | 1 | 8.3 |  | 1 | 8.3 |  | 1 | 8.3 |
